# Sequence Diversity Diagram for comparative analysis of multiple sequence alignments

**DOI:** 10.1186/1753-6561-8-S2-S9

**Published:** 2014-08-28

**Authors:** Ryo Sakai, Jan Aerts

**Affiliations:** 1 Department of Electrical Engineering (ESAT) STADIUS Center for Dynamical Systems, Signal Processing and Data Analytics, KU Leuven, Kasteelpark Arenberg 10, 3001 Leuven, Belgium; 2 iMinds Medical IT, Leuven, Belgium

## Abstract

**Background:**

The sequence logo is a graphical representation of a set of aligned sequences, commonly used to depict conservation of amino acid or nucleotide sequences. Although it effectively communicates the amount of information present at every position, this visual representation falls short when the domain task is to compare between two or more sets of aligned sequences. We present a new visual presentation called a Sequence Diversity Diagram and validate our design choices with a case study.

**Methods:**

Our software was developed using the open-source program called Processing. It loads multiple sequence alignment FASTA files and a configuration file, which can be modified as needed to change the visualization.

**Results:**

The redesigned figure improves on the visual comparison of two or more sets, and it additionally encodes information on sequential position conservation. In our case study of the adenylate kinase lid domain, the Sequence Diversity Diagram reveals unexpected patterns and new insights, for example the identification of subgroups within the protein subfamily. Our future work will integrate this visual encoding into interactive visualization tools to support higher level data exploration tasks.

## Background

The sequence logo [1] has been the most adopted graphical representation for the multiple sequence alignments of nucleotides or amino acids. Its popularity among biologists stems from its simplicity and accuracy in visually communicating the motif or signature of aligned sequence by contrasting the conserved and diverse positions by the height of single letter codes. It emphasizes the most conserved positions effectively, but this visual encoding falls short for comparative analysis of multiple groups of aligned sequences.

In order to compare two sets of multiple sequence alignments, for instance the adenylate kinase lid (AKL) domain of Gram-negative bacteria and Gram-positive bacteria, it requires three figures for analysis: a sequence logo for across all organisms and one for each subfamily. Although each sequence logo efficiently represents and summarizes the amount of information present at every position, it requires high cognitive load to scan back and forth between figures to identify key positions that are shared or differ between two subfamilies. In addition, the sequential frequency of consecutive positions is missing in the traditional sequence logo.

In this paper, we introduce a new visual for presenting multiple sequence alignments for comparative analysis to aid the domain expert in comparing two or more sets of sequence alignments. We set three objectives for the redesign: improving the visual comparison of two sets of aligned sequences, encoding the sequential conservation, and reducing the visual noise. We discuss the strengths and weakness of the sequence logo for the comparative aligned sequence analysis. We also elaborate on our choices for visual encoding and validate our design choices with a case study of the AKL domain dataset, provided through the BioVis 2013 redesign contest. We conclude with our future work to develop an interactive visualization tool for explorative data analysis of multiple sequence alignments.

### Design rationale

A sequence logo figure consists of a stack of letters, representing an amino acid residue or a nucleotide with varying heights to encode the information content of the residue at every position. The information content, also known as the Shannon Entropy [2], is a measure of the uncertainty in a random variable. In the case of sequence logos, this uncertainty is interpreted as how well residues are conserved at each position. For the amino acid sequence alignment, the residues are typically grouped by their structure and the chemical characteristics of their R groups and each residue is color-coded according to the assigned group. In order to compare two groups of aligned sequences, three separate figures are generated: one that combines both samples and one for each group. Although the sequence logo representation may be effective for individual subfamilies, it is not designed for comparing multiple alignment sets.

**Figure 1:**
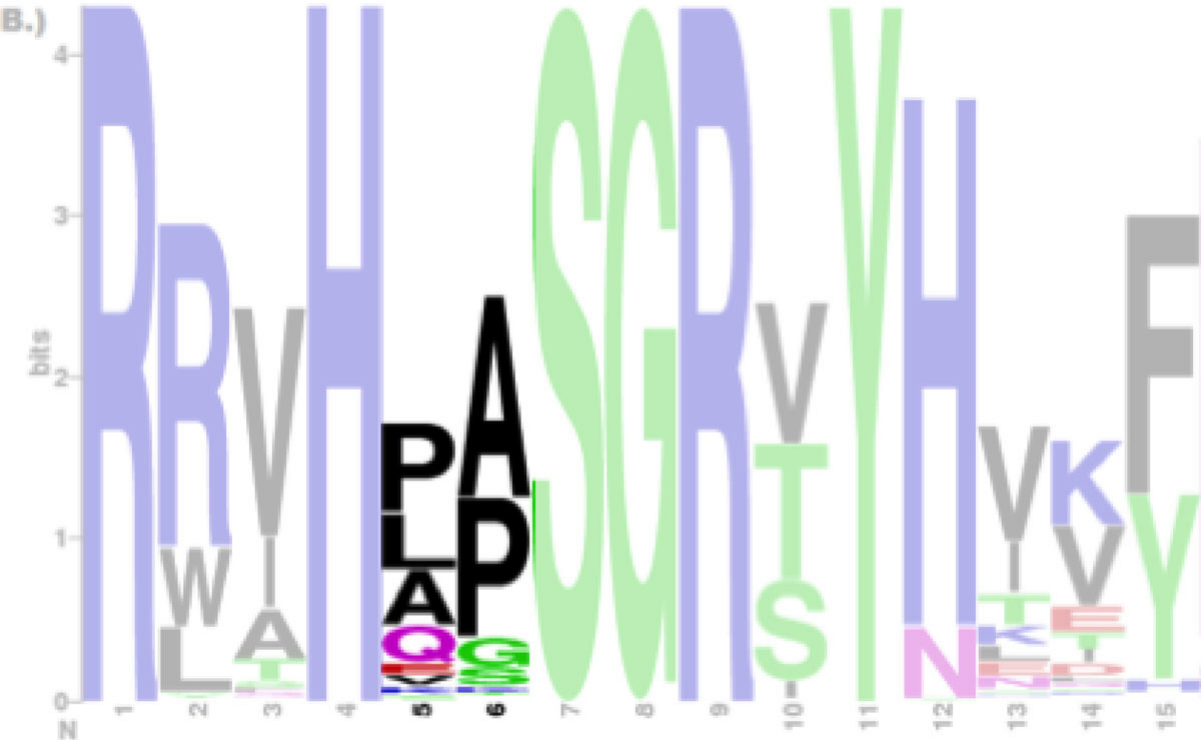
**Sequence logos of the adenylate kinase lid domain from Gram-negative bacteria**. It is not clear whether the Proline (P) at position 5 is followed by the Alanine (A) or the Lysine (K) at subsequent position 6. Only positions from 1 through 15 are shown and masked except for position 5 and 6.

Another motivation for improving on the traditional sequence logo is to encode the sequential information of conserved positions. For example, the Proline (P) and the Lysine (L) at position 5 and Alanine (A), and Proline (P) at position 6 are the most conserved amino acid residues [Figure 1], but the fact that the Proline (P) is followed by the Alanine (A) and the Lysine (L) is followed by the Proline (P) is not clear from this figure. Thus, conveying and communicating the sequential conservation of adjacent and distant residues may aid domain experts to gain new insights or reveal unexpected patterns as it helps to analyze the sequence alignment as sequence, rather than individually by position.

### Related works

**Figure 2:**
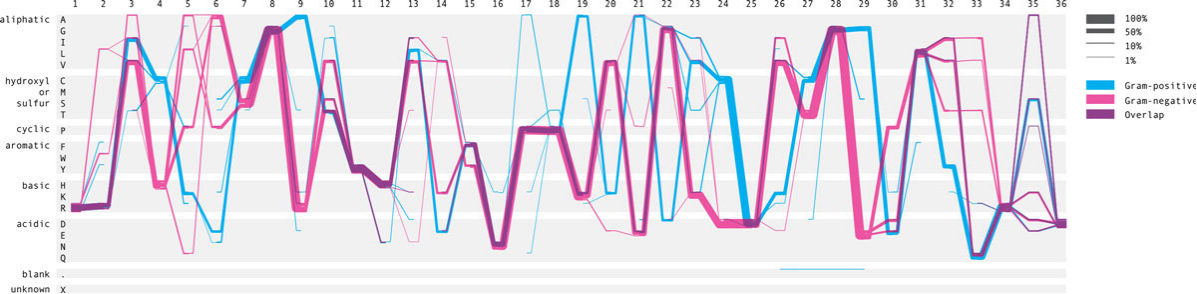
**Parallel Sets representation of the adenylate kinase lid domain**. Both Gram-positive and Gram-negative sequence positions are laid horizontally on the x-axis. Two sets of sequence alignment are separated as categorical data, as shown in the first dimension.

The new work was inspired by the Parallel Sets (ParSet) [3] and the ProfileGrids [4]. ParSet is an interactive visualization application for multi-dimensional categorical data. For each dimension, each of its categories is connected to a number of categories in the subsequence dimension, showing the subdivision of categories. When this visual encoding is applied to the multiple sequence alignment data [Figure 2], it encodes the sequential frequencies, but the functional grouping of amino acids is lost and each vertical bar, representing a frequency at the position, needs to be labeled to indicate which amino acid it is.

ProfileGrids use a visualization which reduces alignments to a matrix, colored according to the residue frequency at each position. Although this visual encoding provides a concise overview of alignments, it does not encode the sequential frequency and still requires multiple matrixes for the comparative analysis.

Sankey diagrams [5] are a type of flow diagram, commonly used to visualize energy or material flows between processes within a system, where the thickness of the arrows is proportional to the quantity. One advantage of using the Sankey diagram over the Parallel Set is that the line or arrow thickness is consistent between dimensions, thus minimizing the effect of the line width illusion [6].

Jalview is an interactive visualization tool for editing, analysis, and annotation of multiple sequence alignments [7]. It is a comprehensive tool which can work with sequence annotation, secondary structure information, phylogenetic trees and three-dimensional molecular structures. Its multiple alignment views allow the same data to be viewed independently in many different ways at the same time, but it requires more than one panel to compare multiple samples.

## Methods

### Implementation

An open-source, java application developed in Processing [8] is available for Linux, Mac OS × and Windows. It loads FASTA files of multiple sequence alignments and a configuration file with visualization parameters, such as grouping and coloring schemes. The aligned set of not only protein sequences, but also nucleotides can be visualized by modifying the configuration file. The application also allows interactive edits to the image and can export PDF or PNG images. The application, source code, and wiki are available at https://bitbucket.org/biovizleuven/sequencediversitydiagram.

## Results

### Visual encoding

**Figure 3:**
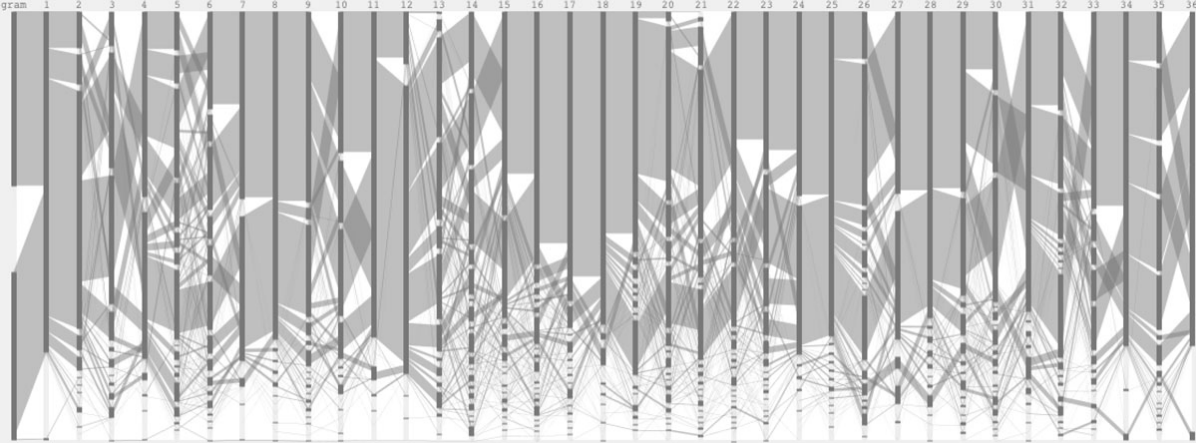
**Sequence diversity diagram of the adenylate kinase lid domain**. The sequence positions are laid horizontally on the x-axis and the residue groupings are shown on the y-axis. The Sequence Diversity Diagrams represent two samples, color-coded and overlaid on top of each other. The line weight represents the relative frequency of residues at the two consecutive positions with respect to the total number of sequences.

We introduce a new visual encoding, called a Sequence Diversity Diagram, for comparative analysis of multiple sequence alignments [Figure 3]. The amino acid positions are plotted horizontally on the x-axis. Amino acid residues and their groupings are plotted on the y-axis. The grouping of amino acids based on their structure and the general chemical characteristics is visually enhanced with horizontal gaps. On this grid layout, a flow diagram is drawn to represent sequence alignment frequency and each sample is color-coded. The line weight represents the relative frequency of residues at the two consecutive positions with respect to the total number of sequences. By linking two adjacent positions, it visualizes the sequential frequency and co-occurrence between two adjacent positions. By overlaying two samples, both the conserved and diverse positions are studied in a single figure.

### Case study

The Sequence Diversity Diagram [Figure 3] is generated from the multiple sequence alignments of the adenylate kinase lid (AKL) domain of Gram-positive and Gramnegative bacteria, provided by the BioVis 2013 contest. By overlaying two sets of sequence alignments and color-coding each, a figure representing both multiple sequence alignments is generated. The positions conserved in both samples are shown in purple [for instance, amino acid 11] and positions that differentiate two subfamilies are found where the lines are not overlapping [amino acid 24]. Less frequent pairs of positions, less than one percent of the total number of aligned sequence in this figure, are filtered out to improve the readability and to reduce the visual noise via the user interface interaction.

The redesigned figure encodes the sequential frequency, and it becomes clear that the Proline at position 5 is followed by the Alanine at position 6. Encoding the sequential frequency also helps to identify subgroups, and another example is a subgroup of Gram-positive from amino acid 19. This subgroup may appear to be "out of phase" oscillations due to a single amino acid insertion or deletion. However, it is unlikely that this is an phasing error, because these subgroups do not share an exact repeated motif even with a phase offset, and the input data are curated structural alignments.

## Conclusions

The Sequence Diversity Diagram is designed to improve the task of visual comparison between multiple sets of aligned sequences, such as protein subfamilies. This Sankey-like diagram is plotted on a grid layout of positions and amino acid functional groups. Instead of having separate sequence logo figures, it consolidates multiple sets of sequence alignments into a single figure. It also encodes the sequential conservation, which may lead to new insights or unexpected findings, such as the identification of the subgroup in the Gram-positive bacteria.

The case study with the AKL domain data demonstrates that this visual encoding is useful for comparative analysis and the result has encouraged us to develop interactive features to further support data exploration tasks. Our future work includes calculation of the mutual information to detect the dependency of one position on another [9]. Then, linking these co-evolving position pairs to the physical proximity or the structural conformation in 3D by loading a protein data bank (PDB) file in Jmol [10]. The aim is to support explorative data analysis at the level of sequence alignment, information theory, and three-dimensional structure. Lastly, we plan to develop a plug-in for widely used applications for multiple sequence alignment analysis, such as JalView, and a reusable BioJS component [11].

## List of abbreviations

AKL -- Adenylate Kinase Lid

PDB -- Protein Data Bank

## Competing interests

The authors declare that they have no competing interests.

## Authors' contributions

RS wrote the software and manuscript under the guidance of JA.
